# The relationships between biological novel biomarkers Lp‐PLA_2_ and CTRP‐3 and CVD in patients with type 2 diabetes mellitus

**DOI:** 10.1111/1753-0407.13574

**Published:** 2024-06-26

**Authors:** Yanhong Chen, Shixin Wang, Jian Li, Yu Fu, Pengsheng Chen, Xuekui Liu, Jiao Zhang, Li Sun, Rui Zhang, Xiaoli Li, Lingling Liu

**Affiliations:** ^1^ Department of Clinical Laboratory Xuzhou Central Hospital Xuzhou China; ^2^ Central Laboratory Xuzhou Central Hospital Xuzhou China; ^3^ Department of Endocrinology Xuzhou Central Hospital Xuzhou China; ^4^ Xuzhou Institute of Medical Science Xuzhou China; ^5^ Department of Cardiology Xuzhou Central Hospital Xuzhou China

**Keywords:** biomarkers, C1q/tumor necrosis factors‐associated protein‐3, cardiovascular disease, lipoprotein‐associated phospholipase A_2_, type 2 diabetes mellitus

## Abstract

**Background:**

Cardiovascular disease (CVD) is recognized as a primary and severe comorbidity in patients with type 2 diabetes mellitus (T2DM) and is also identified as a leading cause of mortality within this population. Consequently, the identification of novel biomarkers for the risk stratification and progression of CVD in individuals with T2DM is of critical importance.

**Methods:**

This retrospective cohort study encompassed 979 patients diagnosed with T2DM, of whom 116 experienced CVD events during the follow‐up period. Clinical assessments and comprehensive blood laboratory analyses were conducted. Age‐ and sex‐adjusted Cox proportional hazard regression analysis was utilized to evaluate the association between lipoprotein‐associated phospholipase A_2_ (Lp‐PLA_2_), C1q/tumor necrosis factor‐related protein 3 (CTRP‐3), and the incidence of CVD in T2DM. The diagnostic performance of these biomarkers was assessed through receiver operating characteristic (ROC) curve analysis and the computation of the area under the curve (AUC).

**Results:**

Over a median follow‐up of 84 months (interquartile range: 42 [32–54] months), both novel inflammatory markers, Lp‐PLA_2_ and CTRP‐3, and traditional lipid indices, such as low‐density lipoprotein cholesterol and apolipoprotein B, exhibited aberrant expression in the CVD‐afflicted subset of the T2DM cohort. Age‐ and sex‐adjusted Cox regression analysis delineated that Lp‐PLA_2_ (hazard ratio [HR] = 1.007 [95% confidence interval {CI}: 1.005–1.009], *p* < 0.001) and CTRP‐3 (HR = 0.943 [95% CI: 0.935–0.954], *p* < 0.001) were independently associated with the manifestation of CVD in T2DM. ROC curve analysis indicated a substantial predictive capacity for Lp‐PLA_2_ (AUC = 0.81 [95% CI: 0.77–0.85], *p* < 0.001) and CTRP‐3 (AUC = 0.91 [95% CI: 0.89–0.93], *p* < 0.001) in forecasting CVD occurrence in T2DM. The combined biomarker approach yielded an AUC of 0.94 (95% CI: 0.93–0.96), *p* < 0.001, indicating enhanced diagnostic accuracy.

**Conclusions:**

The findings suggest that the biomarkers Lp‐PLA_2_ and CTRP‐3 are dysregulated in patients with T2DM who develop CVD and that each biomarker is independently associated with the occurrence of CVD. The combined assessment of Lp‐PLA_2_ and CTRP‐3 may significantly augment the diagnostic precision for CVD in the T2DM demographic.

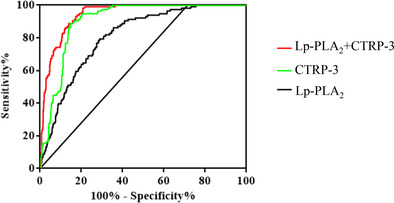

## Graphical abstract

Highlights

- In this study, the positive group of CVD in T2DM showed higher Lp‐PLA_2_ activity and lower CTRP‐3 levels for a 7‐year median follow‐up compared with the negative group.
- The results of this study suggest Lp‐PLA_2_, CTRP3, and their combination detection can effectively increase the diagnostic efficacy of CVD in patients with T2DM, and it will be conducive to further risk assessment of patients with T2DM and predict the high‐risk population of CVD.

## 1 INTRODUCTION

Over the preceding four decades, notable advancements have been made in healthcare delivery and intensive care modalities. However, cardiovascular disease (CVD) remains the predominant cause of hospital admissions and mortality among individuals diagnosed with type 2 diabetes mellitus (T2DM). Indeed, CVD patients with T2DM demonstrate an elevated risk of cardiovascular morbidity and mortality relative to nondiabetic individuals, engendering significant public health concerns and imposing considerable economic burdens.^1^, ^2^, ^3^ T2DM stands as a critical risk factor for CVD, and the clinical presentations and prognoses of CVD are notably more adverse among diabetic patients.^4^, ^5^, ^6^, ^7^

Lipoprotein‐associated phospholipase A_2_ (Lp‐PLA_2_), a constituent of the A_2_ phospholipase superfamily and also known as platelet‐activating factor (PAF) acetylhydrolase, is characterized by a distinctive Gly‐Xaa‐Ser‐Xaa‐Gly sequence commonly associated with lipases. This enzyme exists in at least two isoforms: one intracellular and the other circulating within the plasma. PAF is implicated in the modulation of inflammation, potentially through mechanisms involving PAF‐stimulated enhancement of vascular permeability.^8^, ^9^ Empirical evidence has identified Lp‐PLA_2_ as a vascular‐specific inflammatory marker of significant prognostic value in the assessment of vascular dysfunction.^10^, ^11^, ^12^ Current research posits that an upregulation of Lp‐PLA_2_ expression in atherosclerosis may contribute to the initiation and progression of plaque instability. Furthermore, Lp‐PLA_2_ levels may reflect the degree of atherosclerotic plaque instability and the severity of coronary artery disease.^13^ Huang et al. reported a positive correlation between plasma Lp‐PLA_2_ concentrations and major adverse cardiovascular events in patients experiencing acute myocardial infarction, which intimates a potential role for Lp‐PLA_2_ in the pathogenesis and progression of myocardial infarction.^14^ Nevertheless, the correlation between Lp‐PLA_2_ and CVD in T2DM warrants further rigorous investigation.

C1q/tumor necrosis factor‐related protein 3 (CTRP‐3) is a novel member of the C1q/tumor necrosis factor superfamily and exhibits extensive homology with adiponectin.^15^ The anti‐inflammatory, antioxidative, and antiapoptotic properties of CTRP‐3 have been documented to confer substantial protective effects against CVDs in several studies.^16^ CTRP‐3 is involved in various physiological functions, including the regulation of blood glucose levels, mitigation of inflammation and arteriosclerosis, and facilitation of angiogenesis. CTRP‐3 is also known to suppress the differentiation and proliferation of adventitial fibroblasts through the ADIPOR1/PAMPK/Akt signaling pathway, influencing pathological vascular remodeling.^17^, ^18^ Beyond its physiological roles, CTRP‐3 assumes considerable importance in the etiology and progression of obesity, inflammation, metabolic syndrome, and CVDs.^19^, ^20^, ^21^, ^22^

This investigation is aimed at elucidating the association between Lp‐PLA_2_ activity and CTRP‐3 concentrations and CVD events, specifically, cardiovascular or cerebrovascular mortality, acute coronary syndromes, and coronary stent implantation, in patients with T2DM. Our goal is to ascertain the prognostic value of Lp‐PLA_2_ and CTRP‐3 for CVD in this patient population and to evaluate their potential clinical applicability.

## 2 MATERIALS AND METHODS

### 2.1 Study groups

We performed a retrospective cohort study that was approved by the Ethics Committee of Xuzhou Central Hospital (XZXY‐LJ‐2015‐109‐077) and written informed consent was obtained from each participant or their relatives. T2DM patients who attended the Xuzhou Central Hospital from January 2016 to December 2022 served as the study subjects. The diagnosis of T2DM is based on the 1999 World Health Organization (WHO) criteria. Inclusion criteria were age ≥18 years with the presence of T2DM. The diagnosis of CVD was based on the diagnostic criteria of “Guidelines for rapid diagnosis and treatment of acute coronary syndrome in emergency department.”^23^ The cardiobrovascular diseases and chronic kidney disease, liver infectious disease (creatinine >2 mg/dL or ALT >2 times upper normal limit, respectively), malignant tumor, and any kind of auto‐immune disease were ruled out. The diagnosis of T2DM conformed to the international criteria 2010 issued by the WHO.^24^ The diagnostic criteria of hypertension were defined as follows: systolic blood pressure (SBP) ≥ 140 mmHg (1 mmHg = 0.133 kPa) or diastolic blood pressure (DBP) ≥ 90 mmHg, and it should be measured three times to calculate the average value, or taking antihypertensive drugs.^25^ Dyslipidemia was defined as follows: triglyceride (TG) ≥150 (mg/dL), low‐density lipoprotein cholesterol (LDL‐C) ≥130 (mg/dL), or high‐density lipoprotein cholesterol (HDL‐C) <40 (mg/dL).^26^ The endpoint of this study was the cardiovascular or cerebrovascular death, first occurrence of CVD (mainly includes: cardiovascular death, acute coronary syndrome, coronary stent implantation) or last visit to Xuzhou Central Hospital during the follow‐up period.

**FIGURE 1:**
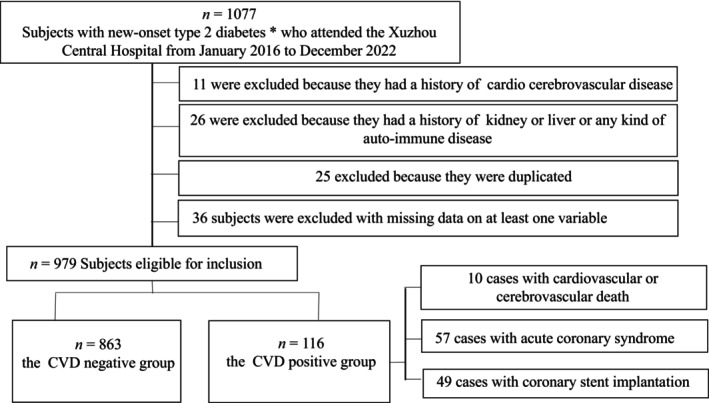
Flow chart of the study population. *The diagnosis of T2DM conformed to the international criteria 2010 issued by the World Health Organization (WHO). CVD, cardiovascular disease (cardiovascular death, acute coronary syndrome, and coronary stent implantation.

A total of 1077 subjects with new‐onset T2DM who attended the Xuzhou Central Hospital from January 2016 to December 2022 were included for the analyses. Eleven cases were excluded because they had a history of cardio‐cerebrovascular disease, 26 cases were excluded because they had a history of kidney or liver or any kind of auto‐immune disease, 25 cases were excluded because they were duplicated, 36 cases of subjects were excluded with missing data on at least one variable. Finally, 979 T2DM patients aged 57.0 (52.0–61.0) years were covered in this study, including 645 males and 334 females. Ultimately, the CVD of T2DM were 10 cases with cardiovascular death (8.6%), 57 cases with acute coronary syndrome (49.2%), and 49 cases with coronary stent implantation (42.2%) (Figure 1). Patients who died of cardiovascular did not experience other events such as coronary syndrome, or stent implantation before admission.

### 2.2 Database and data extraction

The data included patients' demographic and clinical characteristics, age, sex, ethnicity, education, smoking history, alcohol drinking, medical history, previous medications, anthropometric data, blood content, and so forth. Venous blood samples were collected immediately after admission (fasting blood glucose was measured on an empty stomach in the early morning of the second day after admission). Blood samples were collected from the elbow vein, and 4 mL of gel‐containing yellow cap vacutainer was collected, after centrifugation within 1 h (1000*g*, 5 min), the serum was divided into two parts. One part was used to detect total cholesterol (TC), TG, HDL‐C, LDL‐C, apolipoprotein A‐I (ApoA‐I), apolipoprotein B (ApoB), and lipoprotein(a) (Lp(a)), and small and dense particles (sdlDL‐C) were measured by standard enzymatic procedures on an automated chemical analyzer (7600‐020; Hitachi, Tokyo, Japan). Another batch was stored in −80°C refrigerator for measuring CTRP3 concentration and Lp‐PLA_2_ activity. Routine laboratory data such as fasting plasma glucose (FPG), TC, and TG were collected within 5 h. During the follow‐up period, samples collected were tested every 6 months for CTRP3 and Lp‐PLA_2_ data. Glycated hemoglobin A1c (HbA1c) concentration was measured by high‐pressure liquid chromatography (Bio‐Rad Inc, Hercules, CA, USA). The neutrophil gelatinase‐associated lipocalin (NGAL) concentration was determined by chemiluminescence immunoassay kit (CLIA MQ60 plus; Hotgen Biotech Co., Ltd., Beijing). High‐sensitivity CRP (hsCRP) level was tested by particle‐enhanced immunoturbidimetric assay (Hitachi 917 analyzer: Boehringer Mannheim, Germany). CTRP3 was detected by enzyme‐linked immunosorbent assay (ELISA), the kit was purchased from R&D Systems, USA, and the detection method of Lp‐PLA_2_ is rate method, and the kit source is Changchun Hengxiao Biotechnology Co., LTD. The experiment was performed manually following the instructions.

### 2.3 Statistical analysis

All statistical analyses were conducted after the end of the follow‐up period. Continuous variables were reported as either mean and standard deviation or median and interquartile range in accordance with their distribution (assessed through the Shapiro–Wilk test). Quantitative data consistent with normal distribution were compared by using *t*‐test and one‐way analysis of variance was performed between multiple groups, and LSD test was employed for further pairwise comparisons. Mann–Whitney nonparametric test was performed for analysis between two groups for skewed data, Kruskal–Wallis test was performed for analysis between three groups, and the Nemenyi method was adopted for further pairwise comparisons. Univariable Cox regression analysis was performed to identify the significant predictors of the study outcomes among the clinically relevant parameters and the measured data. Next, Cox proportional hazards models were adopted by forcing into the multivariable analysis clinically relevant baseline covariates and biomarkers that were univariably significantly correlated with the primary composite outcome. The receiver operating characteristic (ROC) analysis was also performed to investigate the sensitivity and specificity of the above‐mentioned models, which were undertaken as the predictors of CVD in T2DM. The Youden Index is a measure of the truthfulness of screening tests and represents the overall ability of screening methods to detect real patients versus nonpatients. The higher the Youden Index, the better the effect of the screening experiment and the greater the authenticity. The sensitivity, specificity, and diagnostic efficacy of each index were compared, and the area under the ROC curve (AUC) was calculated by drawing the ROC curve. A two‐sided *p*‐value of less than 0.05 indicated a difference with statistical significance. All statistical analysis was conducted through the SPSS 25.0 software (IBM, USA).

## 3 RESULTS

### 3.1 Baseline characteristics

This study covered 979 subjects, including 645 males (65.9%) and 334 females (34.1%), with an average age of 57.0 (52.0–61.0) years. All subjects were diagnosed with T2DM and the longest follow‐up period was 84 months (42 [32 ~ 54]). During the follow‐up, depending on the occurrence of CVD, the patients were divided into CVD‐positive and ‐negative groups. The description of the two groups' baseline characteristics was summarized in Table 1.

**TABLE 1:** Clinical, biochemical, and angiographic characteristics of study subjects in CVD negative and positive groups in T2DM.

Characteristic	Total	CVD		*p* value
		Negative (*n* = 863)	Positive (*n* = 116)	
Age (years old)	57.0 (52.0–61.0)	57.0 (50.0–61.0)	57.0 (53.0–61.0)	0.967
Male/Female	645/334	568/295	77/39	0.905
BMI (kg/m^2^)	24.7 (22.8–27.5)	24.6 (22.8–27.5)	24.9 (22.8–27.5)	0.383
Alcohol drinking, *n* (%)	571/979 (58.3)	502/863 (58.2)	69/116 (59.5)	0.788
Smoking, *n* (%)	529/979 (54.0)	465/863 (53.9)	64/116 (55.2)	0.793
Hypertension, *n* (%)	475/979 (48.5)	419/863 (48.6)	56/116 (48.3)	0.956
Dyslipidemia (%)	794/979 (81.1)	699/863 (81.0)	95/116 (81.9)	0.816
Antihypertensive therapy, *n* (%)	386/979 (39.4)	350/863 (40.6)	36/116 (31.0)	**0.049**
Statin medication, *n* (%)	580/979 (59.2)	525/863 (60.9)	58/116 (50.0)	**0.026**
Antihyperglycemic drugs, *n* (%)				
Insulin	359/979 (36.7)	311/863 (36.0)	48/116 (41.4)	0.262
Other AHAs	790/979 (80.7)	699/863 (81.0)	91/116 (78.4)	0.514
Metformin	619/979 (63.2)	542/863 (62.8)	77/116 (66.4)	0.453
Sulfonylurea	520/979 (53.1)	462/863 (53.5)	58/116 (50.0)	0.474
Thiazolidinedione	250/979 (25.5)	218/863 (25.3)	32/116 (27.6)	0.590
Glinides	599/979 (61.2)	527/863 (61.1)	72/116 (62.1)	0.835
Glitazones	443/979 (45.3)	389/863 (45.1)	54/116 (46.6)	0.764
α‐glucosidase inhibitor	380/979 (38.8)	335/863 (38.8)	45/116 (38.8)	0.996
DDP‐4i	109/979 (11.1)	96/863 (11.1)	13/116 (11.2)	0.979
SBP (mmHg)	136 (120–139)	136 (119–139)	136 (120–138)	0.445
DBP (mmHg)	86 (74–95)	86 (74–97)	89 (76–92)	0.263
FPG (mg/dL)	117.6 (113.4–125.5)	117.5 (113.3–125.0)	118.1 (114.4–131.0)	0.088
HbA1c (%)	6.5 (5.5–7.4)	6.5 (5.5–7.4)	6.6 (6.7–7.4)	0.419
Insulin (mU/L)	12.5 (9.3–14.6)	12.4 (9.3–14.5)	13.1 (9.4–15.6)	0.085
TG (mg/dL)	123.8 (98.4–154.7)	123.8 (98.5–152.5)	124.4 (98.3–163.6)	0.416
TC (mg/dL)	206.3 (174.4–245.6)	206.2 (175.5–245.6)	206.3 (166.9–245.7)	0.649
HDL‐C (mg/dL)	44.8 (36.0–51.9)	45.0 (36.2–52.4)	42.9 (35.1–47.9)	**0.011**
LDL‐C (mg/dL)	112.7 (91.3–127.2)	111.4 (90.3–126.9)	115.3 (98.2–129.1)	**0.029**
sdlDL‐C (mg/dL)	26.5 (19.6–31.3)	26.6 (19.8–31.1)	26.4 (18.8–32.2)	0.885
ApoA‐I (mg/dL)	134.3 (117.4–147.4)	134.3 (117.1–147.6)	136.6 (120.3–144.5)	0.861
ApoB (mg/dL)	91.6 (79.9–103.2)	91.1 (79.5–102.7)	96.0 (84.0–107.7)	**0.013**
Lp (a) (mg/dL)	18.2 (11.6–32.3)	17.8 (11.6–31.4)	20.5 (13.3–37.4)	0.061
hsCRP (mg/L)	7.6 (5.3–9.2)	7.5 (5.1–9.4)	7.9 (6.8–9.0)	0.102
Neutrophils (×10^9^/L)	3.61 (2.89–4.34)	3.62 (2.91–4.34)	3.54 (2.79–4.38)	0.674
NGAL (μg/L)	89.5 (65.8–111.4)	88.6 (66.1–108.6)	94.5 (61.3–136.1)	0.072
Lp‐PLA_2_ (U/L)	199.8 (149.9–269.9)	189.6 (143.2–251.2)	304.9 (242.3–366.1)	**<0.001**
CTRP‐3 (μg/L)	89.3 (67.6–110.7)	94.8 (75.6–112.8)	59.5 (52.0–64.7)	**<0.001**

*Note*: Variables with *p* < 0.05 were identified as significant variables and they are in bold.

Abbreviation: AHAs, antihyperglycemic agents.

CVD patients of the positive group in T2DM showed significantly increased the levels of LDL‐C (*p* = 0.029), ApoB (*p* = 0.013), Lp‐PLA_2_ (*p* < 0.001), on the contrary, the levels of HDL‐C (*p* = 0.011) and CTRP‐3 (*p* < 0.001) and the proportion of antihypertensive therapy (*p* = 0.049) and lipid‐lowering therapy (*p* = 0.026), were significant lower in positive group compared with negative group (Table 1). Moreover, no significant differences were found in age, gender, body mass index (BMI), smoking or not, alcohol drinking or not, hypertension, dyslipidemia, SBP, DBP, FPG, HbA1c, insulin, TG, TC, sdlDL‐C, ApoA‐I, Lp(a), hsCRP, neutrophils, NGAL between the two groups (*p* > 0.05, respectively; Table 1). In this study, oral hypoglycemic agents covered insulin, other antihyperglycemic agents, metformin, sulfonylurea, thiazolidinedione, glinides, glitazones, α‐glucosidase inhibitor, and DDP‐4i, we have made a corresponding analysis on the use of the above‐mentioned drugs and outcomes of interest. Unfortunately, the above‐mentioned drugs have no differences between the two groups (*p* > 0.05, respectively; Table 1).

### 3.2 Relationships between Lp‐PLA_2_ and CTRP‐3 and risk of CVD of T2DM patients

In this study, Lp‐PLA_2_ and CTRP‐3 with CVD in T2DM were assessed in Cox regression analysis. The occurrence of CVD in T2DM patients and follow‐up time were the dependent variables, whereas age, gender, BMI, smoking, alcohol drinking, hypertension, dyslipidemia, SBP, DBP, HbA1c, insulin, TG, TC, HDL‐C, LDL‐C, sdIDL‐C, Apo‐I, ApoB, Lp(a), hsCRP, neutrophils, NGAL, Lp‐PLA_2_, and CTRP‐3 were set as independent variables. The results showed that the proportion of antihypertensive therapy (hazard ratio [HR] = 0.702, [95% confidence interval {CI}: 0.474–1.041], *p* = 0.078), lipid‐lowering therapy (HR = 0.659 [95% CI: 0.458–949], *p* = 0.037), high levels of LDL‐C (HR = 1.003 [95% CI: 1.000–1.007], *p* = 0.000), ApoB (HR = 1.011 [95% CI: 1.001–1.021], *p* = 0.024), Lp‐PLA_2_ (HR = 1.009 [95% CI: 1.008–1.011], *p* < 0.001), and lower level of HDL‐C (HR = 0.981 [95% CI: 0.964–0.998], *p* = 0.030), CTRP‐3 (HR = 0.936 [95% CI: 0.926–0.946], *p* < 0.001) were independent risk factors for CVD in T2DM (Table 2). In addition, besides HDL‐C, LDL‐C, and ApoB, Lp‐PLA_2_ and CTRP‐3 covered into multivariate Cox proportional models and the results demonstrated that the abnormally expressed Lp‐PLA_2_ activity (HR = 1.007 [95% CI: 1.005–1.009], *p* < 0.001), CTRP‐3 (HR = 0.943 [95% CI: 0.935–0.954], *p* < 0.001) and ApoB (HR = 1.010 [95% CI: 1.001–1.019], *p* = 0.028) were significantly correlated with the occurrence of CVD in T2DM (Table 2).

**TABLE 2:** Cox proportional hazards regression model was used to analyze the risk factors of the occurrence of CVD in T2DM (HR, 95% CI).

Variables	Univariate analysis	Multivariate analysis
Antihypertensive therapy	0.702 (0.474–1.041)*	0.662 (0.438–1.001)*
Lipid‐lowering therapy	0.659 (0.458–0.949)**	0.715 (0.490–1.044)*
HDL‐C	0.981 (0.964–0.998)**	0.983 (0.965–1.002)*
LDL‐C	1.003 (1.000–1.007)*	1.003 (1.000–1.007)*
ApoB	1.011 (1.001–1.021)**	1.010 (1.001–1.008)**
Lp‐PLA_2_	1.009 (1.008–1.011)***	1.008 (1.006–1.010)***
CTRP‐3	0.936 (0.926–0.946)***	0.943 (0.933–0.954)***

*Note*: −: no next step analysis.

* *p* > 0.05;

** *p* < 0.05;

*** *p* < 0.01.

### 3.3 Predictive value of Lp‐PLA_2_ and CTRP‐3

**FIGURE 2:**
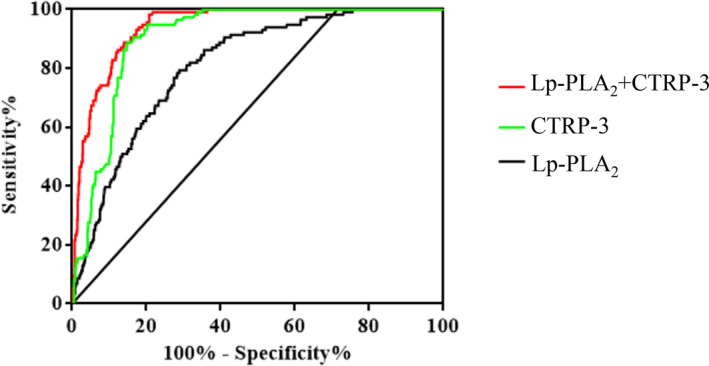
Receiver operating characteristic (ROC) analysis on the predictive capacity of the created multivariate Cox regression model for the occurrence of CVD in patients with T2DM aged 40–65 years. ROC analysis on the predictive capacity of the Lp‐PLA_2_ (AUC = 0.81, 95% CI: 0.77–0.85, *p* < 0.001) and CTRP‐3 (AUC = 0.91, 95% CI: 0.89–0.93, *p* < 0.001) for the identification of the hazard for the primary composite outcome of CVD: Cardiovascular disease (cardiovascular death, acute coronary syndrome and coronary stent implantation) with a sensitivity of 78.45% and 71.61% and a specificity of 94.83% and 79.72%, respectively. Moreover, the combination of Lp‐PLA_2_ and CTRP‐3 improved the prediction capability (AUC = 0.94, 95% CI: 0.93–0.96, *p* < 0.001).

The discriminative capability of Lp‐PLA_2_ and CTRP‐3 was assessed using receiver‐operator characteristic (ROC) curves to determine CVD in T2DM. In all subjects with CVD, the AUC for Lp‐PLA_2_ was 0.81 [95% CI: 0.77–0.85], *p* < 0.001, the optimal cut‐off value of Lp‐PLA_2_ for CVD detection in this study was>238.4 U/L (sensitivity of 78.45%, specificity of 71.61%, Youden Index: 50.06). And the AUC for CTRP‐3 was 0.91 [95% CI: 0.89–0.93], *p* < 0.001, the optimal cut‐off value of CTRP‐3 for CVD detection in this study was <71.7 μg/L (sensitivity of 94.83%, specificity of 79.72%, Youden Index: 74.55). Moreover, the combination of Lp‐PLA_2_ and CTRP‐3 enhanced the prediction capability (AUC = 0.94 [95% CI: 0.93–0.96], *p* < 0.001) (Figure 2 and Table 3).

**TABLE 3:** ROC analysis on the predictive capacity of Lp‐PLA2 and CTRP‐3 for the occurrence of CVD in T2DM.

Variable	AUC	SE	*p*	95% CI	Sensitivity (%)	Specificity (%)	Youden index
Lp‐PLA_2_ + CTRP‐3	0.94	0.01	<0.001	0.93–0.96	98.28	79.18	77.46
Lp‐PLA_2_	0.81	0.02	<0.001	0.77–0.85	78.45	71.61	50.06
CTRP‐3	0.91	0.01	<0.001	0.89–0.93	94.83	79.72	74.55

Abbreviation: SE, standard error.

## 4 DISCUSSION

This present study demonstrated that the levels of Lp‐PLA_2_ activity and CTRP3 levels were abnormally expressed in CVD patients in T2DM. Detection of Lp‐PLA_2_, CTRP3, and their combination can effectively improve the diagnostic efficacy of CVD in patients with T2DM, and help to further evaluate the risk of CVD in T2DM patients and predict the high‐risk population of CVD with T2DM.

CVD is one of the major risk factors for disability or death in T2DM, and inflammation plays an important role in the occurrence and development of T2DM patients who developed CVD.^27^ Lp‐PLA_2_ hydrolyzes acetyl and oxidized residues from the sn‐2 position of PAF and oxidized phospholipids. Lp‐PLA_2_, through its hydrolysate lysophosphatidylcholine (LPC) and nonesterified fatty acids, upregulates the expression of adhesion molecules in endothelial cells, thus inducing the activation of inflammatory factors, activates more monocytes/macrophages to enter the plaque, and can also activate proteolytic enzymes, accelerates rupture of fibrous plaque.^28^ Subsequently, the inflammatory cells in the arteriosclerosis plaque produce more Lp‐PLA_2_,^29^ which forms a positive feedback loop for inflammatory cells and Lp‐PLA_2_ formation in the arterial intima, leading to self‐reinforcing inflammatory responses and acceleration of arterial lesions.^30^ Lp‐PLA_2_ also exerts an anti‐inflammatory action by degrading the pro‐inflammatory mediator PAF and the oxidized phospholipids. Levels of PAF and oxidized phospholipids are elevated in inflammatory conditions leading to higher activity of Lp‐PLA_2_ in order to degrade them. Therefore, Lp‐PLA_2_ is a marker of inflammatory conditions but its role as an independent risk factor is uncertain.^31^ CTRP3, as an adipokine, plays an important role in the regulation of glucose and lipid metabolism and inflammation. Many studies have shown that CTRP3 plays a protective role in cardiovascular system by inhibiting inflammation, relieving oxidative stress, and promoting angiogenesis. Multiple scholars have demonstrated in animal experiments that CTRP3 can promote angiogenesis in the infarct marginal zone by activating signaling pathways such as AKT‐HIF1α‐VEGF, improving blood perfusion, and inhibiting the expansion of the infarct zone.^32^, ^33^ Existing research has revealed that the abnormal expression of Lp‐PLA_2_ and CTRP3 in the serum of patients with coronary heart disease (CHD) exists,^34^, ^35^, ^36^, ^37^ but the regularity and diagnostic value of Lp‐PLA_2_ and CTRP3 in the progression of CVD should be determined in depth. The monitoring and changes of the above‐mentioned new inflammatory factors during the occurrence and development of CHD are also the key breakthrough for us to find CVD early and improve the prognosis of patients with T2DM. In addition, this study suggested that traditional lipid markers such as ApoB (HR = 1.010 [95% CI: 1.001–1.019], *p* = 0.028) remained statistically significant after adjustment for traditional risk factors, which is consistent with previous reports.^38^ Our study found that the proportion of lipid‐lowering therapy was lower in the CVD‐positive group and changes in lipoprotein lipid composition could affect the data on Lp‐PLA_2_ activity, while statin therapy has also been reported to reduce Lp‐PLA_2_ activity, meanwhile, LDL levels were reduced in parallel, statins were less effective at lowering ApoB, and the major structural protein found in LDL, which is consistent with previous reports.^39^, ^40^, ^41^

In this study, we analyzed the relationship between traditional risk factors, new inflammatory modulators Lp‐PLA_2_, CTRP3, and the occurrence of CVD in T2DM. The results showed that the level of serum Lp‐PLA_2_ activity in patients of CVD in T2DM patients was significantly higher than that in CVD‐negative patients, which was positively correlated with the onset of CVD. This effect was still statistically significant after age‐ and sex‐adjusted the traditional risk factors (HR = 1.007 [95% CI: 1.005–1.009], *p* < 0.001). However, the expression of CTRP3 in CVD positive group was significantly lower than that in negative group, which was negatively correlated with the onset of CVD. This effect was still statistically significant after adjusting the traditional risk factors (HR = 0.943 [95% CI: 0.935–0.954], *p* < 0.001), that is to say, CTRP3 is the relative protective factor of CVD of T2DM. The above‐mentioned results will be helpful for the risk assessment of CVD patients and provide clues for predicting the high‐risk population of CVD in T2DM. There is also a trend of early onset, which may be related to diet and lifestyle factors, it is therefore more important to develop biomarkers for CVD onset in T2DM. This study suggested that abnormal Lp‐PLA_2_ activity and levels of CTRP‐3 were significantly correlated with CVD in T2DM. When both Lp‐PLA_2_ and CTRP‐3 were covered in Cox regression analysis, Lp‐PLA_2_ (HR = 1.007 [95% CI: 1.005–1.009], *p* < 0.001) and CTRP‐3 (HR = 0.943 [95% CI: 0.935–0.954], *p* < 0.001) were independently correlated with the risk of CVD in T2DM patients, which was largely consistent with the above results. In addition, as indicated by the result of the ROC curve analysis, Lp‐PLA_2_ can accurately predict the occurrence of CVD in T2DM (AUC = 0.81 [95% CI: 0.77–0.85], *p* < 0.001); CTRP‐3 can be a strong predictor of CVD in T2DM (AUC = 0.91 [95% CI: 0.89–0.93], *p* < 0.001); the joint detection and prediction ability of the two was improved greatly (AUC = 0.94 [95% CI: 0.93–0.96], *p* < 0.001). A total of 238.4 U/L and 71.7 μg/L were determined as the optimal cut‐off points of Lp‐PLA_2_ and CTRP‐3 to predict the risk of CVD in T2DM patients, with a sensitivity of 78.45% and 71.61% and a specificity of 94.83% and 79.72%, respectively. The findings in this study could affect the featuring lipid or chronic inflammation biomarkers profiling as a useful tool in routine clinical practice in T2DM at high risk of CVD. To help clinicians to further risk stratification of T2DM, the development and validation of new biomarkers is particularly important, this not only provides additional data support for traditional clinical and laboratory data, but also facilitates the creation of personalized predictions and treatments, thus expanding the precise concept of CVD based on T2DM in daily clinical work.

## 5 CONCLUSIONS

In conclusion, this study confirmed that the serum Lp‐PLA_2_ activity and levels of CTRP3 were abnormally expressed in CVD patients of T2DM, the measurement of Lp‐PLA_2_ and CTRP‐3 could improve the diagnostic efficacy for the detection of patients prone for CVD in T2DM. And it will be conducive to further risk assessment of patients with T2DM and predict the high‐risk population of CVD in T2DM. Therefore, it can be said that monitoring Lp‐PLA_2_ and CTRP3 in patients with T2DM will be helpful to early warning and diagnosis of CVD, and take on clinical significance in improving the prognosis of the disease.

## 6 LIMITATIONS

It is undeniable that there are some limitations in this study, which should be improved by further experiments. First, the data of this study are all from our hospital patients, more than 95% of them are Han nationality, sampling error is inevitable. In addition, differences in ethnic group, lifestyle, and clinical use of drugs may lead to bias in the analysis. Next, the testing kit was relatively single and was implemented by a single laboratory rather than a multicenter study, and the grouping of subjects was based solely on the results of clinical examinations in different departments, instead of a comprehensive assessment of the proportion and extent of disease onset. Thus, the sample size, multicenter, and large sample size validation should be further expanded. In addition, this study aimed to compare the differences in Lp‐PLA_2_ and CTRP3 levels between patients with or without CVD in T2DM, to identify the potential CVD population at an early stage, thus achieving early intervention and improved disease outcomes, no further subgroup analysis was performed in CVD patients (distinguishing between cardiovascular death, acute coronary syndrome, and, coronary stent implantation), this study will be focused on in future research. Finally, the characteristics of CHD are gender‐specific, with higher morbidity and mortality in men than in women,^42^ a phenomenon also reflected in the sex ratio of the study population (less than 35% of female patients), considering that men and women may differ regarding risk factors for the same disease, in the next study we plan to distinguish between sex subgroups after expanding the sample size and to analyze disease risk factors separately. So far, there is no obvious breakthrough in the interaction between Lp‐PLA_2_ and CTRP3 in the process of CVD in T2DM patients, which is one of the key points to be studied next.

## AUTHOR CONTRIBUTIONS

Yanhong Chen, Shixin Wang, and Jian Li contributed equally to this work. Yanhong Chen, Shixin Wang, Xiaoli Li, and Lingling Liu contributed to the conception and design of the work, data analysis, interpretation of the data, and manuscript writing; Yu Fu, Pengsheng Chen, Jiao Zhang, and Xuekui Liu collected and analyzed data; Shixin Wang and Li Sun searched and interpreted the data and critically revised the manuscript; and Lingling Liu and Xiaoli Li contributed to the conception and design of the work, acquisition and interpretation of the data, and critical revision of the manuscript. All authors approved the final version of the manuscript for publication and they agreed to be accountable for all aspects of the work. Lingling Liu and Xiaoli Li were the guarantors of this work, and as such, had full access to all the data in the study and took responsibility for the integrity of the data and the accuracy of the data analysis. All authors reviewed the manuscript.

## FUNDING INFORMATION

This study was supported by the Key Research and Development Program of Xuzhou City (grant number KC22153).

## CONFLICT OF INTEREST STATEMENT

The authors have declared no conflicts of interest.

## Data Availability

The datasets used and/or analyzed during this current study are available from the corresponding author upon reasonable request.
